# Inactivation of a *Plasmodium* apicoplast protein attenuates formation of liver merozoites

**DOI:** 10.1111/j.1365-2958.2011.07787.x

**Published:** 2011-08-17

**Authors:** Joana M Haussig, Kai Matuschewski, Taco W A Kooij

**Affiliations:** Parasitology Unit, Max Planck Institute for Infection BiologyCharitéplatz 1, 10117 Berlin, Germany

## Abstract

Malaria parasites undergo a population expansion inside the host liver before disease onset. Developmental arrest inside host hepatocytes elicits protective immune responses. Therefore, elucidation of the molecular mechanisms leading to mature hepatic merozoites, which initiate the pathogenic blood phase, also informs anti-malaria vaccine strategies. Using targeted gene deletion in the rodent model malaria parasite *Plasmodium berghei*, we show that a *Plasmodium*-specific Apicoplast protein plays an important role for Liver Merozoite formation (PALM). While the resulting knockout mutants develop normally for most of the life cycle, merozoite release into the blood stream and the ability to establish an infection are severely impaired. Presence of a signature blood-stage antigen, merozoite surface protein 1 and normal apicoplast morphology indicate that the inability to finalize merozoite segregation is a direct consequence of loss of *PALM* function. Experimental immunization of mice with as few as two doses of *palm*^-^ sporozoites can elicit sterile protection up to 110 days after final immunization. Our data establish that a tailor-made arrest in the final steps of hepatic merozoite formation can induce strong protective immune responses and that malaria parasites employ a distinct apicoplast protein for efficient formation of pre-erythrocytic merozoites.

## Introduction

Malaria parasites are obligate intracellular eukaryotic pathogens that belong to the phylum *Apicomplexa*. *Plasmodium falciparum* is a major cause of childhood mortality in sub-Saharan Africa and continues to be the most prevalent and fatal arthropod-borne infectious disease [World Health Organization (WHO), 2011].

*Plasmodium* species follow a complex developmental programme of stage conversions as they progress through their life cycles (Prudencio *et al*., 2006; Silvie *et al*., 2008a). After transmission through infectious *Anopheles* bites, the motile sporozoites rapidly migrate through the skin and enter a blood capillary (Amino *et al*., 2006). Sporozoites pass to the liver where they adhere to the endothelium of the sinusoid. There, they enter by breaching Kupffer cells and several hepatocytes before finally taking residence in a suitable hepatocyte (Mota *et al*., 2001; Frevert *et al*., 2005). Target cell entry coincides with the formation of a replication-competent niche, termed the parasitophorous vacuole, which originates from the host cell plasma membrane (Sibley, 2011). The parasitophorous vacuole membrane prevents fusion of the developing parasite with endosomal compartments, thus providing a safe environment for liver-stage development. After a few days of extensive growth and replication, thousands of first-generation merozoites are released into the blood stream in vesicles termed merosomes (Sturm *et al*., 2006). After being released in the pulmonary microvasculature (Baer *et al*., 2007), free merozoites quickly invade red blood cells, thus initiating a malaria blood-stage infection. The subsequent fast rounds of asexual replication within host erythrocytes allow the parasite to multiply exponentially and are the exclusive cause of the clinical manifestations of malaria (Haldar *et al*., 2007).

A hallmark of apicomplexan parasites is the presence of a vestigial organelle, termed apicoplast, acquired by secondary endosymbiosis of a red alga (Janouskovec *et al*., 2010). The apicoplast contains a 35 kb circular DNA encoding two *rRNA* units, 25 *tRNA* units and 30 mainly housekeeping proteins (Wilson *et al*., 1996). Most of its metabolic activity comes from an estimated 500–600 nuclear-encoded proteins that are imported using a bipartite leader sequence (Waller *et al*., 2000). Although the apicoplast has long since lost its photosynthetic functionality, a number of important functions have been retained, most notably the biosynthesis of fatty acids, haem and isopentenyl diphosphate (Ralph *et al*., 2004). All three pathways differ considerably from those found in the host. Moreover, this unique organelle is indispensable for parasite survival, which has made it one of the leading targets for novel anti-malarial drug development. For instance, fosmidomycin interferes with the nonmevalonate pathway of isoprenoid biosynthesis (Jomaa *et al*., 1999), while several antibiotic agents act as apicoplast translation inhibitors (Dahl and Rosenthal, 2007), thus causing a so-called delayed death phenotype (Fichera and Roos, 1997).

Irradiation-induced arrest of liver-stage maturation induces strong protective immune responses in the host (Nussenzweig *et al*., 1967; Hoffman *et al*., 2010). Using experimental genetics, loss-of-function mutants displaying a defined developmental arrest before parasite replication have been generated. Consecutive delivery of these genetically arrested parasites elicits protection against reinfection (van Dijk *et al*., 2005; Mueller *et al*., 2005a,b; 2007;). Therefore, characterization of *Plasmodium* genes that are important for liver-stage development also informs anti-malaria vaccine development.

More recently, mutants that display an arrest in late liver-stage development have been described. Deficiencies in the type II fatty acid biosynthesis (FASII) pathway lead to premature arrest, as evident from the absence of signature proteins of mature blood-stage infectious parasites (Yu *et al*., 2008; Vaughan *et al*., 2009; Pei *et al*., 2010). Targeted deletion of liver-specific protein 1 (LISP1) leads to formation of hepatic merozoites but a partial defect in parasitophorous vacuole membrane rupture (Ishino *et al*., 2009). Sporozoite-specific ablation of the vital cGMP-dependent kinase revealed that mutant parasites are unable to complete liver-stage development (Falae *et al*., 2010). However, it remains to be shown whether these parasites are still able to finalize merozoite formation and whether they express any blood-stage proteins.

Here, we demonstrate the importance of a previously uncharacterized *Plasmodium*-specific apicoplast protein for liver merozoite formation (PALM) and its requirement for efficient transition to a blood-stage infection.

## Results

### Identification of a candidate *Plasmodium* liver stage-specific protein

**Fig. 1:**
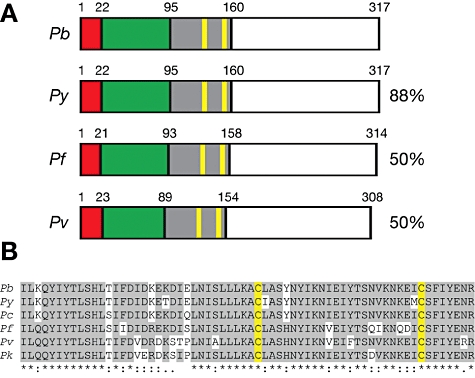
The *Plasmodium*-specific apicoplast protein important for liver merozoite formation (PALM). A. Primary structure of *Plasmodium* PALM proteins. Shown are the overall sequence structures and amino acid sequence identities of PALM orthologues in *P*. *yoelii* (PY01863), *P. falciparum* (PFF0110w) and *P. vivax* (PVX_113280), compared with *P. berghei* PALM (PBANKA_010110). Signal peptide (red), apicoplast-targeting sequence (green) as predicted with PlasmoAP (Foth *et al*., 2003), and the conserved domain (grey) with two signature cysteine residues (yellow) are shown. B. Sequence alignment of *Plasmodium* PALM proteins. Shown is the conserved domain (A) with the two signature cysteine residues (highlighted yellow). *P. berghei* PALM, PBANKA_010110; *P*. *yoelii* PALM, PY01863; *P. chabaudi* PALM, PCHAS_010180; *P. falciparum* PALM, PFF0110w; *P. vivax* PALM, PVX_113280; *P. knowlesi* PALM, PKH_114760.

To identify *Plasmodium* genes with a specific critical function in liver merozoite formation, we systematically searched available data sets for potential liver stage-specific genes of unknown function. We focused on a small *Plasmodium*-specific gene (PBANKA_010110) expressed during liver-stage development (Tarun *et al*., 2008). The only additional expression data available are from a recent RNA sequencing effort of synchronous *P. falciparum* blood stages that revealed negligible PALM transcription levels with a slight upregulation in mature blood stages (Otto *et al*., 2010). Orthologues were readily identified in other *Plasmodium* species but not in any other apicomplexan parasite. Using cross-species comparisons, we manually re-annotated the *Plasmodium berghei* gene and were able to identify a previously unrecognized apicoplast targeting sequence (Fig. 1A). In addition, we noticed a central, highly conserved domain that contains two strictly conserved cysteine residues (Fig. 1A and B).

### PALM is upregulated in *Plasmodium* liver stages and localizes to the apicoplast

**Fig. 2:**
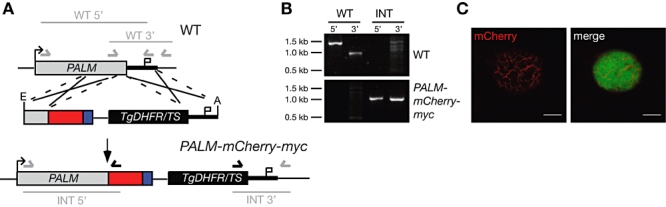
Live cell imaging of PALM in infected hepatoma cells. A. Generation of *PALM-mCherry-myc* parasites. The *PbPALM* genomic locus was targeted with a replacement plasmid containing the C-terminal *PALM* fragment (grey) fused in-frame to the *mCherry* coding sequence (red), and a quadruple *c-myc* tag (blue) and followed by the 3′UTR of *PbDHFS/FPGS*. In addition, the targeting plasmid contains the *PbDHFR/TS* positive selectable marker (black box), and a fragment of the *PALM* 3′UTR. Upon a double cross-over event, the targeting plasmid is expected to replace the endogenous *PALM* ORF with a C-terminally tagged PALM fusion protein. Arrows and bars indicate specific primers and PCR fragments respectively. B. Genotyping of the *PALM-mCherry-myc* parasite line. Using integration-specific primer combinations (A), the successful replacement event was verified. Absence of the WT signal from *PALM-mCherry-myc* parasites confirmed the purity of the clonal population. C. Hepatoma cells were infected with *PALM-mCherry-myc* sporozoites. PALM expression was visualized in late liver stages 2 days after infection. Note the branched structure of the mCherry signal. Bars, 10 µm.

To detect PALM throughout the parasite life cycle, we attempted to raise specific peptide antibodies, but the obtained sera gave non-specific signals (data not shown). As an alternative, we generated a parasite line, using a *P. berghei* ANKA clone constitutively expressing GFP (ANKA-GFP; Janse *et al*., 2006), with the endogenous PALM protein fused to the red fluorescent protein mCherry (Shaner *et al*., 2004) and a quadruple myc tag (Fig. 2A and B). The resulting transgenic *PALM-mCherry-myc* parasites completed the full life cycle without any impairment (Table S1). Comparable with ANKA-GFP, three C57BL/6 mice bitten by three *PALM-mCherry-myc* parasite-infected mosquitoes each became patent on day 4. We first monitored PALM expression in live parasites. We observed structured, albeit weak, signals in *in vitro* cultured liver-stage parasites (Fig. 2C). Absence of a detectable signal in any of the other life cycle stages indicated that PALM expression levels in general are low, with the exception of mature liver stages.

**Fig. 3:**
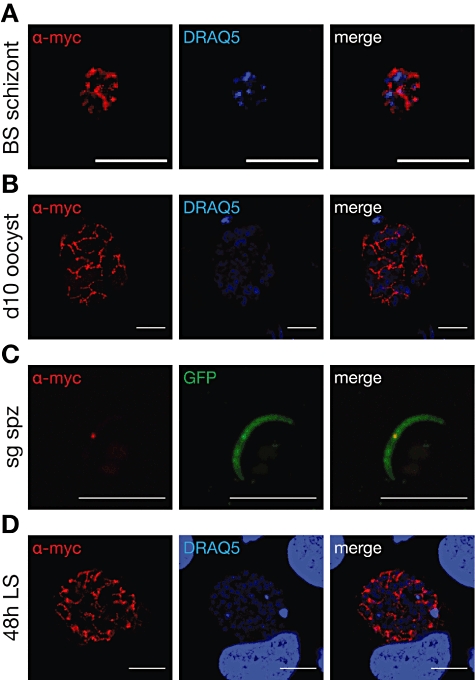
Expression of PALM during the *Plasmodium berghei* life cycle. *PALM-mCherry-myc* parasites were used to infect mice and *Anopheles stephensi* mosquitoes. Intra- and extracellular parasite stages were fixed, permeabilized and stained with mouse anti-myc antibody (red). In replicating stages, nuclei were stained with the DNA-dye DRAQ5 (blue). The GFP signal in fixed sporozoites was used to display the extracellular parasite. A. Blood-stage schizont (BS schizont) from an infected mouse. Note that the anti-myc signal is very weak and was only detectable after background subtraction in comparison with WT parasite-infected erythrocytes. Bars, 5 µm. B. Oocysts fixed at day 10 after infection. Bars, 10 µm. C. Salivary gland sporozoites fixed at day 17 after infection. Bars, 10 µm. D. Liver-stage parasites fixed at 48 h after infection of hepatoma cells. Bars, 10 µm. Note the branched structure indicative of an apicoplast localization of PALM.

We next employed indirect immunofluorescence microscopy using mouse anti-myc antibodies on fixed parasites, to take advantage of the quadruple myc epitope (Fig. 3). In good agreement with the report of low levels of *PALM* transcripts in mature blood stages (Otto *et al*., 2010), we were able to detect a structured PALM signal in mature asexual blood stages but not in ring stages or trophozoites. Moreover, we observed a structured staining inside developing *Plasmodium* oocysts that segregated in mature oocysts, as well as a single dot in salivary gland sporozoites, indicative of the apicoplast. These findings are in good agreement with the previously unrecognized apicoplast targeting signal (Fig. 1A).

**Fig. 4:**
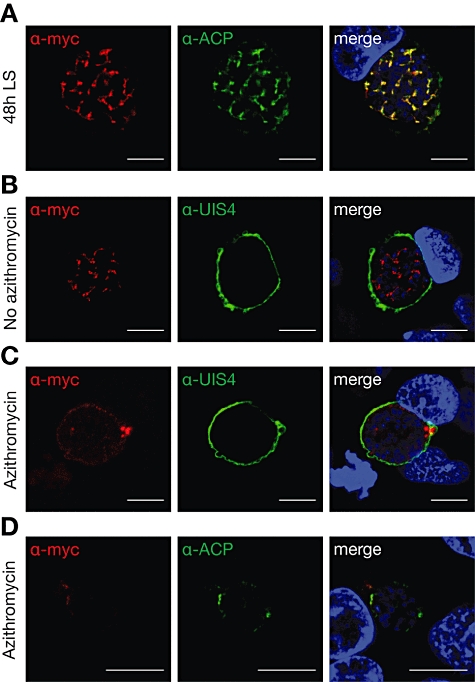
Apicoplast localization of PALM. A. Co-staining of fixed, *PALM-mCherry-myc* parasite-infected hepatoma cells 48 h after infection using anti-myc and anti-ACP antibodies. Note the substantial overlap between PALM and the signature apicoplast protein. B. *PALM-mCherry-myc* parasite-infected hepatoma cells left untreated, fixed at 48 h after infection, and stained with antibodies specific for the myc epitope and upregulated in infectious sporozoites protein 4 (UIS4), a signature protein of the parasitophorous vacuole. Note the branched PALM-positive structures. C. Antibiotics treatment with 1 µM azithromycin abolishes the branched PALM-positive structures while parasites appear to remain healthy otherwise. D. Antibiotics treatment with 1 µM azithromycin abolishes the branched PALM-positive structures as well as ACP-positive structures. All bars, 10 µm.

To further corroborate localization of PALM to this vestigial organelle, we focused on liver stages where the PALM signal is most prominent. We used polyclonal antibodies against a signature apicoplast protein, acyl carrier protein (ACP; Friesen *et al*., 2010), and co-stained fixed *PALM-mCherry-myc* parasite-infected hepatocytes with monoclonal anti-myc antibody (Fig. 4A). The staining overlapped substantially, strongly suggesting that tagged PALM is indeed transported efficiently into the apicoplast.

In apicomplexan parasites, the apicoplast and the mitochondrion are in close association (Tonkin *et al*., 2004), necessitating additional proof for an exclusive localization to one of the two organelles. Previous work established that antibiotic treatment during liver-stage development abrogated apicoplast growth and segregation (Friesen *et al*., 2010). We therefore repeated the immunolocalization experiment in the presence or absence of 1 µM azithromycin (Fig. 4B–D). Destruction of the apicoplast integrity by this treatment abolished the ACP- and PALM-positive branched structure and, instead, limited the signal to a small peripheral dot. Together, our data show that *PALM* is expressed in blood-stage schizonts, throughout the parasite's mosquito stage development, and, most abundantly, during liver-stage development. In all stages, PALM localizes to the apicoplast of the developing parasite.

### Blood and mosquito stage development of *palm*^-^ parasites is unaffected

**Fig. 5:**
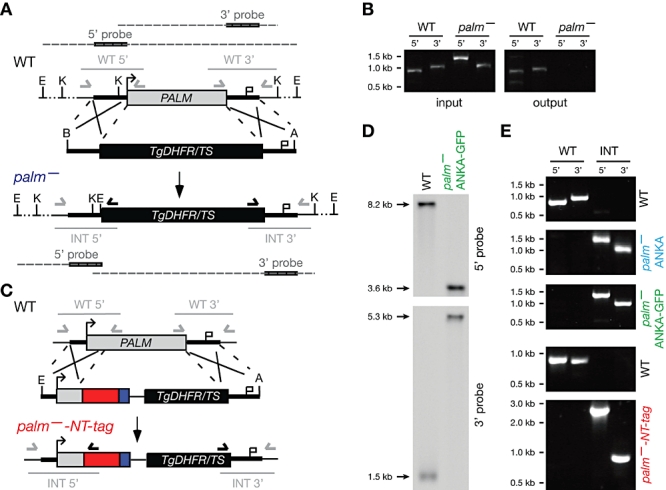
Generation of *palm*^-^ parasites. A. The *PbPALM* genomic locus was targeted with a replacement plasmid containing *PALM* 5′ and 3′ fragments (thick black lines) and the *DHFR/TS* positive selectable marker (black box). Upon a double cross-over event, the targeting plasmid is expected to replace the endogenous *PALM* ORF. Arrows and light grey solid lines indicate specific primers and PCR fragments respectively. Black bars indicate specific 5′ and 3′ probes; dark grey dashed lines represent EcoRI or KpnI restriction-digested fragments of WT or knockout parasite gDNA. B. Mouse-mosquito-mouse transmission experiment starting from a mixed WT/*palm*^-^ infection (input) and resulting in a pure WT population after completion of one transmission cycle (output). Parasites were genotyped by PCR using wild-type (WT)- and replacement (INT)-specific primer pairs, as indicated in (A). C. Alternative gene replacement vector to ablate the *PALM* locus. The *PbPALM* genomic locus was targeted with a replacement plasmid containing a *PALM* 5′ fragment, containing a portion of the PALM open reading frame (grey box), fused to the mCherry protein (red box) and quadruple myc tag (blue box), followed by the *DHFR/TS* positive selectable marker (black box) and the 3′ flanking region (thick bar). Upon a double cross-over event, the targeting plasmid is expected to replace the endogenous *PALM* ORF. Arrows and bars indicate specific primers and PCR fragments respectively. D. Genotyping of the *palm*^-^ ANKA-GFP parasite line used for the majority of experiments through Southern blot analysis. Probes at the 5′ and 3′ UTR of *PALM* were used to demonstrate the expected size shift of restriction-digested gDNA of WT and knockout parasites. E. Genotyping of the three clonal *palm*^-^ parasite lines obtained from independent transfection experiments. Using integration-specific primer combinations (A, C) the successful replacement event (INT) was verified. Absence of the WT signal from *palm*^-^ parasites confirmed the purity of the clonal populations. The clonal populations *palm*^-^ ANKA (blue) and *palm*^-^ ANKA-GFP (green) were obtained according to design (A), using WT-ANKA and ANKA-GFP parasites as recipient lines (left); the clonal population *palm*^-^*-NT-tag* (red) was obtained according to design (C), using ANKA-GFP parasites as recipient lines (right).

Our analysis in conjunction with available *PALM* expression data suggested a minor role during replication of asexual blood stages. We therefore attempted to ablate PALM by targeted gene deletion using a replacement strategy through homologous recombination (Fig. 5A). After transfection of wild-type ANKA strain parasites and positive selection, the recombinant parental parasite population was genotyped (Fig. 5B). Replacement-specific PCR products indicated successful deletion of *PALM* in our recombinant parental parasite population.

In order to test whether *PALM* performs a vital role during *Plasmodium* life cycle progression, we first tested the mixed parental population, containing both ANKA wild type and *palm*^-^ ANKA parasites, in a mouse-mosquito-mouse passage (Fig. 5B). Only ANKA parasites were detectable after completion of the life cycle, suggestive of a vital role at some point during transmission between mammalian hosts.

To systematically characterize *PALM* loss-of-function parasites, we generated two additional, independent mutant parasites that lack *PALM*. To generate the second *palm*^-^ Iine, we used the same targeting vector as above (Fig. 5A) but transfected fluorescent parasites, so-called ANKA-GFP parasites (Janse *et al*., 2006). The resulting parasites were labelled *palm*^-^ ANKA-GFP. In a third transfection experiment, we used a slightly modified targeting vector that was predicted to replace PALM with a fusion protein of the N-terminal PALM apicoplast targeting sequence to the red fluorescent protein mCherry and a quadruple myc-tag (Fig. 5C). This line, termed *palm*^-^*-NT-tag*, represents a functional gene knockout and a targeting reporter construct, simultaneously.

From each of the three independent transfections, we generated clonal *palm*^-^ parasite lines by *in vivo* cloning through limited dilution. Genotyping revealed the presence of the predicted integration events (Fig. 5D and E). In the following sections, the majority of the presented data were generated using the *palm*^-^ ANKA-GFP line. This was possible, as careful phenotyping of the three independent *palm*^-^ lines revealed identical behaviour under all assay conditions (Table S1), indicating that the results obtained from one line can be attributed directly to loss of *PALM* function.

Successful ablation of the *PALM* gene in asexual blood stages already indicated a dispensable role during the pathogenic phase of the *Plasmodium* life cycle. Indeed, *palm*^-^ ANKA-GFP parasites grew without any obvious difference compared with ANKA-GFP parasites and reached equally high parasitaemia levels in infected mice (Fig. S1A).

We next transmitted all three *palm*^-^ clones and the *PALM-mCherry-myc* parasites, as an additional WT-like reference line, to the mosquito vector and followed sporogony in the female *Anopheles* host (Fig. S1 and Table S1). Compared with ANKA-GFP parasites, no differences in numbers of either infected mosquitoes (Fig. S1B) or sporozoites in the mosquito midguts or salivary glands were detectable (Fig. S1C). In conclusion, *PALM* is dispensable for *Plasmodium* blood-stage development, transmission to the mosquito vector, and sporogony in the invertebrate host.

### *palm*^-^ parasites are severely impaired in initiation of a blood-stage infection

**Fig. 6:**
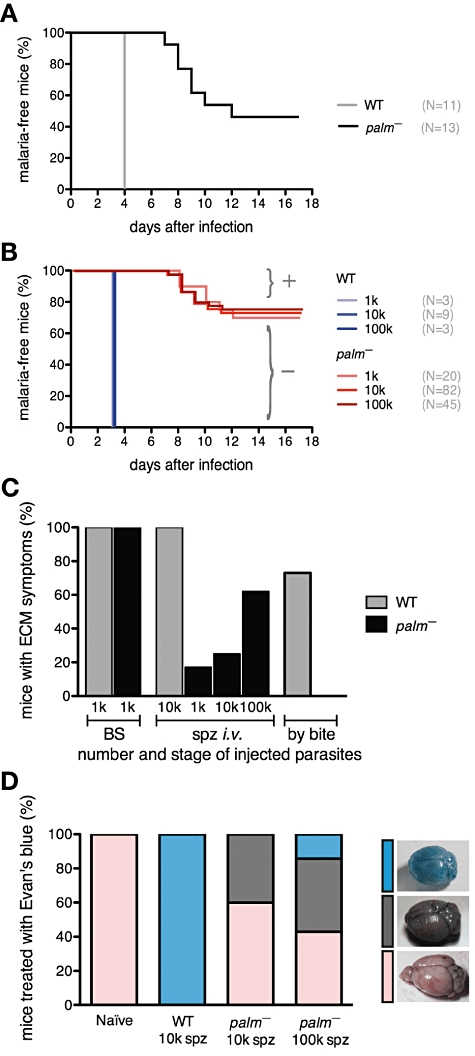
Infection with *palm*^-^ sporozoites leads to attenuated liver-stage development *in vivo*. A. Kaplan–Meier analysis of time to malaria blood-stage infection. C57BL/6 mice were infected by natural bite with five mosquitoes infected with ANKA-GFP (grey, *n* = 11) or *palm*^-^ ANKA-GFP (black, *n* = 13). Animals were monitored daily for presence of parasites in Giemsa-stained blood smears. B. Kaplan–Meier analysis of time to malaria blood-stage infection. C57BL/6 mice were infected with 1000, 10 000, or 100 000 ANKA-GFP (blue, *n* = 3, 9, 3) or *palm*^-^ ANKA-GFP (red, *n* = 20, 82, 45) sporozoites by intravenous injection. Animals were monitored daily for presence of parasites in Giemsa-stained blood smears. C. Development of symptoms of ECM is only reduced in *palm*^-^ sporozoite-infected animals. Shown is the percentage of parasite-positive C57BL/6 mice that develop signature symptoms of ECM (i.e. mice showing sudden onset of ataxia, paralysis, convulsion or coma, or a minimum of three behavioural and functional abnormalities) after injection of 1000 blood-stage parasites (ANKA-GFP, grey, *n* = 15; *palm*^-^ ANKA-GFP, black, *n* = 15), injection of sporozoites [10 000 ANKA-GFP, grey, *n* = 9; *palm*^-^ ANKA-GFP, black, 1000 (*n* = 6), 10 000 (*n* = 20), or 100 000 (*n* = 13)], or infection by natural bite with five mosquitoes infected with ANKA-GFP (grey, *n* = 11) or *palm*^-^ ANKA-GFP (black, *n* = 13). D. Integrity of the blood–brain barrier is preserved in most *palm*^-^ sporozoite-infected mice. Mice were infected with 10 000 ANKA-GFP sporozoites (*n* = 4), or 10 000 (*n* = 5) or 100 000 (*n* = 7) *palm*^-^ ANKA-GFP sporozoites. Evan's blue was injected intravenously at day 4 after appearance of blood-stage parasites and brains removed for documentation. One mouse infected with 100 000 *palm*^-^ ANKA-GFP sporozoites showed behavioural abnormalities and breakdown of the blood–brain barrier.

Normal colonization of the *Anopheles* salivary glands (Fig. S1C) permitted mosquito-to-mouse transmission experiments. When we fed five infected mosquitoes on naïve C57BL/6 mice, ∼50% of mice bitten by *palm*^-^ GFP-ANKA-infected mosquitoes remained completely malaria-free, while the other half showed a severe delay in patency ranging from 3 to 8 days (Fig. 6A).

We next inoculated ANKA-GFP or *palm*^-^ sporozoites into C57BL/6 mice by intravenous injection of increasing doses and monitored the occurrence of blood-stage parasites (Fig. 6B). All mice injected with ANKA-GFP sporozoites became patent on day 3 after infection. In sharp contrast, patency of mice injected with sporozoites of either of the three *palm*^-^ lines was severely delayed by at least 4 days. In the rodent malaria model system, a delay of 1 day in patency corresponds to a 10-fold reduction in initial asexual blood-stage parasite load. Most importantly, a large proportion (∼70%) of mice remained entirely malaria-free at day 17 after infection (Fig. 6B). This pre-erythrocytic *in vivo* defect explains the initial observation, based on genotyping, of an important role of *PALM* during the life cycle (Fig. 5B).

Irrespective of the molecular role of *PALM*, the mutant parasite lines are valuable tools to address two important questions in malaria research. First, does a prolonged pre-erythrocytic phase influence the clinical outcome of a sporozoite-induced infection? This problem can be addressed by analysing the mice that become blood-stage-positive (Fig. 6B; population ‘+’). Second, does immunization with *palm*^-^ sporozoites induce protection against reinfection? This question can be addressed by boosting the mice that stay malaria-free (Fig. 6B; population ‘−’) with additional *palm*^-^ sporozoites, followed by WT challenge infections.

### Impaired liver-stage development leads to reduced incidence of experimental cerebral malaria (ECM)

We first followed the clinical symptoms of population ‘+’, i.e. those mice that became *palm*^-^ blood-stage-positive (Fig. 6C). Only few *palm*^-^-infected mice developed symptoms of ECM, a fatal outcome of an acute *Plasmodium* infection, as compared with ANKA-GFP-infected animals. Signature signs of ECM are sudden onset of ataxia, paralysis, convulsion or coma (de Souza *et al*., 2010). Symptoms of ECM typically develop on the fourth day after patency in sporozoite-induced infections. Of note, none of the mice that were infected by bite of *palm*^-^-infected mosquitoes developed ECM.

It is important to exclude any impairment in parasite virulence due to the genetic manipulation. We previously detected normal blood-stage parasite growth (Fig. S1A). To confirm that *palm*^-^ parasites are as virulent as WT parasites; we inoculated 1000 erythrocytes infected with either *palm*^-^ ANKA-GFP or, as controls, ANKA-GFP parasites via blood transfusion, thus bypassing the liver merozoite phase (Fig. 6C). All animals developed the signature ECM symptoms and had to be carefully culled to avoid unnecessary suffering. Therefore, the reduced ECM incidence of *palm*^-^-infected mice can be entirely attributed to the altered pre-erythrocytic development of these parasites.

We finally determined the integrity of the blood–brain barrier in sporozoite-injected animals by Evan's blue stain at the peak of clinical symptoms in WT-infected animals, typically day 4 after patency (Fig. 6D). This analysis further corroborated the strongly reduced cases and severity of ECM-related pathology in the *palm*^-^-positive mice.

### Immunization with *palm*^-^ parasites induces potent protection against reinfection

We next tested whether booster immunization with *palm*^-^ parasites could confer protection against sporozoite challenge in population ‘−’ (Table 1). Extensive previous work with either irradiated sporozoites or genetically arrested parasites showed that in the *P. berghei*-C57BL/6 model three consecutive doses of attenuated parasites are needed to induce sterile immunity (Nussenzweig *et al*., 1967; Mueller *et al*., 2005b; Douradinha *et al*., 2007). In addition, two studies reported sterile protection of between 25% and 70% of mice up to 10 days following immunization using two immunizations (van Dijk *et al*., 2005; Mueller *et al*., 2005a).

**Table 1:** Immunization with *palm*^-^ sporozoites confers potent protection against reinfection

Challenge	*palm*^-^ immunization^a^	# Protected/# Challenged^b^	Prepatency (days)^c^
Exposure to five infected mosquitoes	2 × 10 000 spz	20/20 (100%)	–
	2 × 1000 spz	0/2 (0%)	6.0
	naïve	0/10 (0%)^d^	4.0
Intravenous injection of 10 000 sporozoites	2 × 10 000 spz	5/5 (100%)	–
	naïve	0/6 (0%)	3.0
Intravenous injection of 10 000 sporozoites (day 110)^e^	2 × 10 000 spz	6/7 (86%)	8.0
	naïve	0/3 (0%)	3.0
Intravenous injection of 10 blood-stage parasites^f^	2 × 10 000 spz	0/4 (0%)	6.0
	naïve	0/5 (0%)	6.0

a Age-matched female C57BL/6 mice were immunized with *palm*^-^ sporozoites (spz) in the numbers indicated. The immunization protocol is described in the *Experimental procedures* section.

b Animals were checked for parasitaemia by daily examination of Giemsa-stained blood smears.

c Prepatency is the time until detection of the first parasite in the peripheral blood.

d One additional naïve mouse (not included) stayed initially malaria-free but became positive at day 3 after intravenous re-challenge.

e Challenge at 110 days after the last immunization.

f The immunized mice were previously shown to be fully protected against ‘by bite’ challenge.

Anticipating comparable levels of protection using three immunization rounds, we directly attempted a two-dose immunization protocol, with 10 000 *palm*^-^ ANKA-GFP sporozoites each (Table 1). Intriguingly, this immunization protocol elicited sterile protection in all mice against both intravenous sporozoite challenge and naturally transmitted malaria, i.e. sporozoite delivery by mosquito bites. Even after a period of more than 100 days, six of seven mice were completely protected from reinfection following an initial challenge of 10 000 intravenously injected ANKA-GFP sporozoites. The single mouse that developed a blood-stage malaria infection showed a substantial delay in patency of 5 days, suggestive of the presence of strong pre-erythrocytic immunity, and did not develop any ECM-associated symptoms. When we reduced the immunizations to two doses of 1000 sporozoites sterilizing immunity and protection development of signs of ECM was lost but infections became patent with a 2 day delay, indicative of a substantial degree of protective immune responses.

We also re-challenged four mice that had previously been immunized twice with 10 000 *palm*^-^ ANKA-GFP sporozoites and challenged by ANKA-GFP-infected mosquito bites with ten ANKA-GFP blood-stage parasites. We observed no protection or delay in prepatency in comparison with naïve control mice (Table 1). Together, these findings demonstrate that *palm*^-^ genetically arrested parasites can elicit potent protective liver stage-specific immune responses against reinfection with as few as two immunization doses.

### *palm*^-^ parasites form liver schizonts *in vitro*

**Fig. 7:**
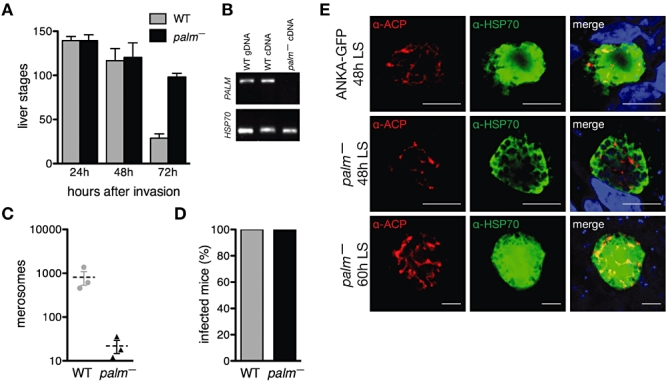
*palm*^-^ parasites display a defect in liver-stage maturation. A. Quantification of liver stages in cultured hepatoma cells at 24, 48 and 72 h after infection with 10 000 *P. berghei* ANKA-GFP and *palm*^-^ ANKA-GFP sporozoites. Infected cells were fixed and stained with mouse anti-*Pb*HSP70 antibodies. Note that in contrast to ANKA-GFP parasites, *palm*^-^ ANKA-GFP parasites remain inside the host cells even at 72 h after infection. Data are from two independent experiments done in triplicate. Shown are mean numbers (± SD). B. Parasite transcript detection in WT and *palm*^-^ parasite-infected livers confirms the successful ablation of *PALM* in the knockout line. cDNA was prepared from liver homogenates extracted from mice at 44 h after sporozoite infection. Quality of cDNA preparations and presence of parasites *in vivo* was controlled using *HSP70*-specific primers. C. Defect of merosome formation in *palm*^-^ parasites. Quantification of merosomes obtained from cultured hepatoma cells infected with ANKA-GFP and *palm*^-^ ANKA-GFP parasites at 72 h after inoculation with 100 000 sporozoites. D. *palm*^-^ ANKA-GFP merosomes are infectious to mice. Complete merosome containing supernatants harvested under (C) were injected into recipient C57BL/6 mice (ANKA-GFP, *n* = 5; *palm*^-^, *n* = 4) and monitored for malaria infections by examination of Giemsa-stained blood-smears. E. Apicoplast morphology appears normal in *palm*^-^ liver stages. Hepatoma cells infected with WT and *palm*^-^ parasites were fixed at 48 or 60 h after infection and stained for parasite cytoplasm and the apicoplast using anti-HSP70 and anti-ACP antibodies respectively. The merge includes an additional DRAQ5 nuclear stain. Bars, 10 µm.

The severe impairment of pre-erythrocytic development *in vivo* together with the unprecedented potent protection obtained by immunization with *palm*^-^ genetically arrested parasites prompted us to analyse the precise developmental arrest of these parasites *in vitro*. We first monitored intra-hepatic parasite growth in cultured hepatoma cells (Fig. 7A). Quantification of liver-stage parasites revealed no differences at 24 and 48 h after infection. Interestingly, we noticed the presence of high numbers of *palm*^-^ liver-stage parasites at 72 h after infection, when ANKA-GFP and *PALM-mCherry-myc* parasites have typically formed merosomes and egressed from the host cells (Fig. 7A). Apparently, more than two-thirds of *palm*^-^ parasites remain intracellular, whereas this is true for less than one-third of ANKA-GFP parasites. This finding offered a first potential explanation for the observed deficiency of *palm*^-^ parasites to complete a full life cycle (Fig. 5B).

At this point, we also wanted to further confirm the absence of *PALM* transcripts in the *palm*^-^ ANKA-GFP parasites by RT-PCR, as late liver stages display the most abundant *PALM* transcription in WT parasites. To this end, we harvested livers from WT- and *palm*^-^-infected mice 44 h after infection and performed RT-PCR on the isolated RNA (Fig. 7B). Absence of *PALM*-specific transcripts further confirmed the successful ablation of the gene, while a signature gene of mammalian parasite stages, *HSP70*, was readily detectable in all livers of either WT- or *palm*^-^-infected animals.

We next harvested and quantified liver merozoite-containing vesicles, termed merosomes, from infected hepatoma cells (Fig. 7C). In contrast to ANKA-GFP parasite-infected cells, we repeatedly failed to obtain substantial merosome numbers from *palm*^-^-infected cells. However, when injected intravenously into naïve NMRI mice, *palm*^-^ ANKA-GFP merosomes were fully infectious and all inoculated mice became eventually malaria-positive (Fig. 7D).

To test whether the apicoplast, the organelle harbouring PALM, is affected by *PALM* ablation, we stained hepatoma cells with *PALM-mCherry-myc* and *palm*^-^ parasites 48 h after sporozoite addition with anti-ACP antibodies (Fig. 7E). As exemplified for the *palm*^-^*-NT-tag* line, mutant liver stages showed no obvious difference when compared with the transgenic control, i.e. *PALM-mCherry-myc*. We conclude that late liver stages appear normal in *palm*^-^ parasites.

### *palm*^-^ parasites display a defect in liver merozoite formation *in vitro*

**Fig. 8:**
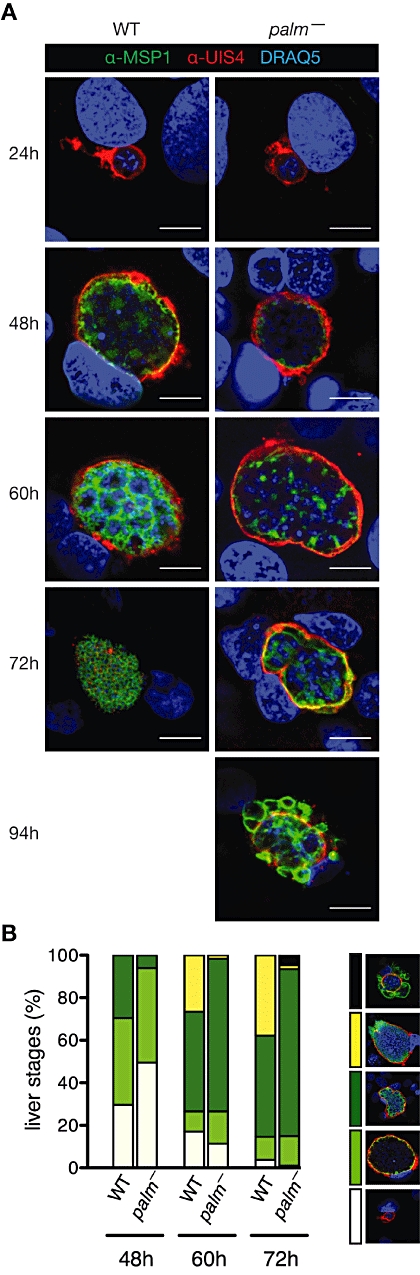
*palm*^-^ parasites cannot finalize liver merozoite formation efficiently. A. Defect in liver-stage merozoite segregation in *palm*^-^ parasites. *In vitro* cultured *P. berghei* ANKA-GFP and *palm*^-^ ANKA-GFP liver stages, were fixed at various time points after sporozoite infection and visualized by immunofluorescence using monoclonal mouse anti-*P. yoelii* MSP1 and rabbit anti-*P. berghei* UIS4 antibodies. Nuclei were stained with the DNA-dye DRAQ5 (blue). *palm*^-^ ANKA-GFP parasites develop normally until late liver stages but rarely form merozoites. Shown are representative images of ANKA-GFP and *palm*^-^ ANKA-GFP at various time points. Bars, 10 µm. B. Quantification of productive liver merozoite formation. Liver stages from ANKA-GFP and *palm*^-^ ANKA-GFP parasites were scored at 48, 60 and 72 h after infection (ANKA-GFP *n* = 105, 94, 82; *palm*^-^*n* = 99, 124, 107) according to the five categories indicated by the representative images in the inset. The categories are: no MSP1 (white), rim staining of MSP1 (light green), intracellular schizont staining of MSP1 (dark green), formation of MSP1-positive merozoites (yellow), and aberrant, external MSP1 staining (black). Note the high proportion of MSP1-positive schizonts in *palm*^-^ parasites, which correlates with a low production of MSP1-positive pathogenic merozoites.

We wanted to characterize the apparent late developmental arrest further and, therefore, stained ANKA-GFP and *palm*^-^ ANKA-GFP parasites during liver-stage development with antibodies against two signature proteins: upregulated in infectious sporozoites protein 4 (UIS4), which localizes to the parasitophorous vacuole (Mueller *et al*., 2005b), and the major merozoite surface protein 1 (MSP1; Holder and Freeman, 1981) (Fig. 8A). In good agreement with successful liver-stage growth (Fig. 7A), ANKA-GFP and *palm*^-^ ANKA-GFP were indistinguishable at 24 and 48 h after infection. At this point in parasite development, ANKA-GFP and *palm*^-^ ANKA-GFP parasites commence MSP1 expression with the protein primarily located to the rim of the developing schizont.

We noticed striking differences when we monitored merozoite formation. Despite normal development until late liver stages, very few *palm*^-^ ANKA-GFP parasites formed merozoites compared with ANKA-GFP parasites (Fig. 8B). At this stage, and even more pronounced at 94 h after infection, numerous *palm*^-^ ANKA-GFP parasites show MSP1 staining outside the parasitophorous vacuole. We conclude that in the absence of *PALM* merozoite segregation is aborted. Together, these findings demonstrate a distinct and important role of *PALM* in full maturation of liver merozoites. In the absence of *PALM*, efficient formation of liver merozoites is the limiting step in *Plasmodium* life cycle progression.

## Discussion

In this study, we characterized a novel apicoplast targeted protein of the malaria parasite with an important function during liver merozoite formation; hence, the term ‘*Plasmodium* apicoplast protein important for liver merozoite formation’ (PALM). PALM loss-of-function parasites retain their capacity to infect and mature inside hepatocytes. However, towards the final steps of merozoite segregation, *palm*^-^ parasites display a strong impairment, resulting in a severe delay in sporozoite-induced blood-stage infections.

### Towards the function of PALM

Unlike several genetically arrested parasites deficient in apicoplast-targeted proteins of the FASII pathway (Yu *et al*., 2008; Vaughan *et al*., 2009; Pei *et al*., 2010), *palm*^-^ parasites develop to a much later time point, as indicated by the expression of the merozoite signature protein MSP1. In this study, we were able to define many key features of this previously unrecognized protein, including the subcellular localization, the life cycle stages at which PALM is expressed, and the critical time point of PALM activity. What could be the molecular function during the process of merozoite formation?

Many proteins imported into the apicoplast perform metabolic functions in one or more of the key processes assigned to this enigmatic organelle. Indeed, applying a recently developed extended similarity group method (Chitale *et al*., 2009), *P. falciparum* PALM is predicted to display potential transferase activity of uncertain specificity, which may be attributed to the conserved domain with the invariant cysteine residues (Fig. 1). However, it is equally conceivable that the cysteine pair fulfils structural or other roles.

Based on the distinct later time point of arrested development in comparison with FASII pathway mutants, a metabolic role in the biogenesis of fatty acids is unlikely. Development of biochemical assays and metabolome profiling, which have not been achieved yet in *Plasmodium*-infected liver cells, would be needed to support this rather speculative prediction. A puzzling question is why PALM is not equally important for other *Plasmodium* life cycle stages, in particular in blood-stage schizonts and during mosquito midgut sporozoite formation, where we have clearly demonstrated the presence of the myc-tagged PALM protein.

Staining of developing liver-stage *palm*^-^ parasites with antibodies against a signature apicoplast-targeted protein (ACP) demonstrated that apicoplast growth, branching and protein import of ACP are unaffected in the absence of *PALM*. We can, therefore, reject the hypothesis that PALM is involved in the process of apicoplast segregation itself. This is plausible, because of the observed expression of PALM in blood-stage schizonts and mosquito stage midgut oocysts and the apparent absence of PALM orthologues in other obligate intracellular parasites of the phylum *Apicomplexa*.

The strictly liver stage-specific role of *PALM in vivo* is further supported by the differential clinical outcome of a blood stage-induced vs. a sporozoite-induced infection. We can exclude a detectable *in vivo* role of *PALM* during blood-stage replication, which in the *P. berghei* ANKA-C57BL/6 mouse model leads to a uniform fatal outcome.

An intriguing possibility is that *PALM* emerged in response to specific requirements for the development of malaria parasites in hepatocytes. Because pre-erythrocytic development of avian malaria species, such as *Plasmodium gallinaceum*, does not occur in hepatocytes but rather in cells of the mononuclear phagocyte system, it is conceivable that these parasites lack *PALM*. In the absence of complete genome sequences of avian *Plasmodium* species, cloning and sequencing of the orthologous region should reveal whether *P. gallinaceum* harbours a *PALM* orthologue or not. The sheer number of merozoites being produced during the pre-erythrocytic phase may require a yet unidentified synthetic pathway in the apicoplast that is dispensable for other life cycle stages and related apicomplexan parasites.

### Delayed pre-erythrocytic development is beneficial for the clinical outcome

Intriguingly, a substantial proportion of those animals infected with *palm*^-^ sporozoites and developing blood-stage parasitaemia do not develop ECM. This finding is reminiscent of the consistent findings seen in phase II clinical trials with the RTS,S vaccine (Guinovart *et al*., 2009; Moorthy and Ballou, 2009). Efficacy of this anti-sporozoite subunit vaccine was particularly robust against severe malaria as endpoint. It was proposed that partial pre-erythrocytic protection, as seen in RTS,S, might translate into sustained asexual stage protection through leakage of low-dose parasites (Guinovart *et al*., 2009). The *palm*^-^ parasites in combination with C57BL/6 mice permit a detailed immunological analysis of a potential link between clinical outcome and the time to emergence and inoculum size of liver-stage merozoites. Therefore, the generation of a potent, yet leaky, late liver stage arrested vaccine line may recapitulate key findings of the candidate malaria vaccine RTS,S/AS, which is tested in phase III clinical trials (Cohen *et al*., 2010). The demonstration of normal virulence by transfusion-mediated infection with *palm*^-^ parasites (Fig. 6C) excludes a direct role of PALM in ECM. Instead, we consider it likely that a prolonged liver-stage infection resulting in a particularly slow onset of blood-stage infection influences the host defence and provides an advantage that prevents the development of ECM symptoms.

### Development of second-generation genetically arrested parasites

As exemplified by the generation of the *palm*^-^ parasites, a tailor-made genetic arrest in liver-to-blood-stage conversion has the potential to elicit potent protective immune responses against future malaria infections. We employed the *P. berghei*-C57BL/6 model, because it is the most stringent rodent malaria model, i.e. the most difficult to protect against (Ngonseu *et al*., 1998; Doolan and Hoffman, 2000). Complete sterilizing immunity protecting all immunized animals by only two doses of genetically arrested *P. berghei* parasites is unprecedented thus far. Two studies have reported protection in a fraction of mice by two consecutive immunizations (van Dijk *et al*., 2005; Mueller *et al*., 2005a). In addition to incomplete protection, in both cases challenges were performed already after 7 or 10 days following the final boost, and in one case the immunization doses were also substantially higher. So far, there is just a single study describing long-term protection of 118 days using the *P. berghei*-C57BL/6 model with genetically arrested *Pbuis3*^-^/*uis4*^-^ parasites (Jobe *et al*., 2007). The protocol employed three subsequent immunization rounds and it is unclear, but unlikely in the light of incomplete short-term protection acquired using *uis4*^-^ parasites, that a single booster would suffice. Development of experimental immunization protocols with lower and fewer doses is particularly important in the context of integrating a safe, affordable and accessible malaria vaccine into the expanded programme of immunization for infants. Although still elusive, such a vaccine should ideally have the capacity to induce potent immune responses after as few postnatal care visits as possible.

It is tempting to speculate that inclusion of additional late liver-stage antigens, such as shown here for abundant expression of MSP1 in *palm*^-^ parasites, in a whole-organism vaccine line may elicit superior immune responses, i.e. lasting protection with fewer and/or lower vaccine doses. There is precedence for potent short- and long-term protection by delivery of low-dose sporozoite inocula under chloroquine cover (Roestenberg *et al*., 2009; 2011;). These results indicate that advanced liver-stage development may correlate with more potent immune responses. This notion is supported by a subunit vaccine strategy using viral vaccine vectors that express one of several *Py*MSP1 fragments (Draper *et al*., 2009). Immunization induced CD8+ T-cell responses that correlated with partial anti-liver-stage protection in the *Plasmodium yoelii*-BALB/c model. Our findings suggest that generation of a safe, MSP1-positive, genetically arrested parasite line may be an important improvement over first generations, which are arrested during onset of liver-stage development. Due to substantial breakthrough infections (Fig. 6B), direct translation of *palm*^-^ parasites as genetically arrested parasites for human vaccine trials is currently not possible. However, additional gene deletions that affect late liver-stage parasite development in the *palm*^-^ background could be used to generate a safe synthetic late-stage arrested parasite.

In conclusion, we describe a previously uncharacterized protein, PALM, which localizes to the apicoplast. We show that PALM plays an important role during the final stages of hepatic merozoite maturation and is required for the efficient transition to a blood-stage infection. Genetic arrest in stage conversion from completion of clinically silent development inside the host liver to pathogenic asexual blood stages has the potential to elicit potent protection against reinfections.

## Experimental procedures

### Experimental animals

This study was carried out in strict accordance with the German ‘Tierschutzgesetz in der Fassung vom 22. Juli 2009’ and the Directive 2010/63/EU of the European Parliament and Council ‘On the protection of animals used for scientific purposes’. The protocol was approved by the ethics committee of the Berlin state authority (‘Landesamt für Gesundheit und Soziales Berlin’, permit number G0469/09). C57BL/6 mice were used for sporozoite challenges and analysis of ECM. All other parasite infections were conducted in NMRI mice, unless otherwise indicated.

### Generation of *PALM-mCherry-myc* and *palm*^-^ parasites

Mutant parasites were generated as described previously (Janse *et al*., 2006). See supporting experimental procedures and Table S2 for further details on transfection vector construction and confirmation of successful transfection.

### *Plasmodium* mosquito stage development

*Anopheles stephensi* mosquitoes were raised under a 14 h light/10 h dark cycle at 28°C and 75% humidity. Blood-feeding and mosquito dissection were performed as described previously (Vanderberg, 1975). In order to determine infectivity, and quantify midgut and salivary gland associated sporozoites, infected mosquitoes were dissected at days 10, 14 and 17 after feeding respectively.

Midguts of *PALM-mCherry-myc* infected mosquitoes were dissected at day 10 and fixed with 4% paraformaldehyde with 0.0075% glutaraldehyde. Subsequently, midguts were permeabilized with 0.5% Triton X-100 and blocked with 3% bovine serum albumin. Incubation with mouse anti-myc antibodies (1:200 dilution, Santa Cruz Biotechnology) was done overnight at 4°C. Bound antibodies were detected using donkey anti-mouse IgG Alexa Fluor 488 conjugated antibodies (1:2000 dilution, Invitrogen). Nuclei were visualized with the DNA-dye DRAQ5 (1:1000 dilution, Axxora) and coverslips were mounted with Fluoromount-G (Southern Biotech).

Salivary glands of *PALM-mCherry-myc* infected mosquitoes were dissected and liberated sporozoites were settled in RPMI medium containing 3% bovine serum albumin and fixed with 4% paraformaldehyde. Subsequently, sporozoites were permeabilized with 0.1% Triton X-100 in PBS and blocked with 3% bovine serum albumin. Incubation with mouse anti-myc antibodies (1:100 dilution, Santa Cruz Biotechnology) was done overnight at 4°C. Bound antibodies were detected using donkey anti-mouse IgG Alexa Fluor 546 conjugated antibodies (1:1000 dilution, Invitrogen). Coverslips were mounted with Fluoromount-G (Southern Biotech).

To determine sporozoite infectivity to mice, sporozoites collected from mosquito salivary glands were injected intravenously at the numbers indicated into C57BL/6 mice. Patency was determined by examination of daily Giemsa-stained thin blood smears.

### Phenotyping of *Plasmodium* life cycle progression

*Plasmodium berghei* mosquito stages were maintained and analysed using standard techniques (Vanderberg, 1975). During all experiments, mice were monitored for the development of behavioural and functional abnormalities (Lackner *et al*., 2006), as indicators of ECM development. Mice were classified as suffering from ECM when they were diagnosed with at least three behavioural and functional abnormalities (e.g. positional passivity, body position, limb grasping, toe pinch, etc.). Mice were immediately sacrificed upon showing sudden onset of signature symptoms of ECM, such as ataxia, paralysis, convulsions or coma. To test the integrity of the blood–brain barrier as a further indication for the susceptibility to ECM, we injected 100 µl of 2% Evans Blue in saline i.v. into naïve C57BL/6 mice, or mice infected with 10 000 or 100 000 *palm*^-^, or 10 000 ANKA-GFP sporozoites at day 4 after patency. After 1 h, mice were sacrificed, brains prepared, and digital images taken using standardized lighting, exposure and white balance settings.

### *Plasmodium* liver-stage development in cultured hepatoma cells

*Plasmodium berghei in vitro* liver stages were cultured and analysed using standard techniques (Silvie *et al*., 2008b). In short, 30 000 hepatoma (HuH7) cells per well were plated in eight-well chamber slides (Nalge Nunc International). After 24 h the cells were incubated with 10 000 sporozoites, first for 60 min at room temperature, then at 37°C for 90–120 min. Non-invaded sporozoites were washed off and medium was changed daily. At the time points indicated, infected hepatoma cultures were fixed for 10 min with ice-cold methanol and blocked with PBS/10% FCS.

For confirmation of expression, liver-stage *PALM-mCherry-myc* parasites were fixed and incubated with mouse anti-myc antibodies (1:1000 dilution, Santa Cruz Biotechnology). To confirm apicoplast targeting of PALM, liver-stage parasites were co-stained with rabbit anti-*P. berghei* ACP peptide antiserum (1:750 dilution; Friesen *et al*., 2010). Completion of liver merozoite formation was analysed using rabbit anti-*P. berghei* upregulated in infectious sporozoites protein 4 (UIS4) peptide antiserum (1:2000 dilution; kindly provided by G. Montagna, MPI-IB, Berlin) and a monoclonal mouse anti-*P. yoelii* merozoite surface protein 1 (MSP1) antibody against a 90 kDa N-terminal fragment of the protein that shares 74% identity with *P. berghei* MSP1 (1:2000 dilution; kindly provided by T. Holder, National Institute for Medical Research, London, UK). Liver-stage parasites were visualized and quantified using monoclonal mouse anti-*P. berghei* heat shock protein 70 (HSP70) antibodies (1:300 dilution; Tsuji *et al*., 1994). Bound antibodies were detected using donkey anti-rabbit/mouse IgG Alexa Fluor 488/546 conjugated antibodies (1:3000 dilution, Invitrogen). Nuclei were visualized with DNA-dyes Hoechst 33342 (Invitrogen) and DRAQ5 (Axxora; both 1:1000 dilution) and coverslips were mounted with Fluoromount-G (Southern Biotech). Images were recorded using a Leica TCS SP-1 confocal microscope. Total numbers of parasites were counted using a Leica DM2500 epifluorescence microscope.

To confirm the apicoplast localization of PALM, we used 1 µM azithromycin (Pfizer) treatment of sporozoite-infected hepatoma cells as described previously (Friesen *et al*., 2010).

Merosome formation was followed using two different methods, either by (i) seeding of 100 000 to 150 000 hepatoma cells per well in 24-well plates and inoculation with 100 000 sporozoites per well 24 h later, or by (ii) seeding of 30 000 hepatoma cells per well in eight-well chamber slides (Nalge Nunc International) and inoculation with 10 000 sporozoites 24 h later. Thereafter, standard procedures were used (Silvie *et al*., 2008b). Merosomes were harvested and counted in a Neubauer chamber 72 h after infection. Infectivity of *in vitro* cultured ANKA-GFP and *palm*^-^ parasites was tested by injection of the complete merosome containing liver-stage culture supernatants in naïve NMRI mice. All animals were monitored for parasitaemia by daily Giemsa-stained thin blood smears.

### Immunization and parasite challenge experiments

Age-matched female C57BL/6 mice were immunized with two doses of 1000 or 10 000 *palm*^-^ sporozoites extracted from salivary glands of infected mosquitoes. Sporozoites were injected intravenously in a volume of 100 µl. For the prime/boost protocol, animals that remained malaria-free after the first immunization were given a second dose 5–7 weeks after the first immunization. Only animals that remained blood-stage parasite-negative after the first immunization and subsequent boost were used for the challenge experiments at 4–6 weeks after the last immunization. Mice were challenged with five ANKA-GFP-infected mosquitoes, 10 000 intravenously injected ANKA-GFP sporozoites, or 10 intravenously injected ANKA-GFP blood-stage parasites. The number of ANKA-GFP salivary gland sporozoites per mosquito used for the challenge by bite ranged from 20 000 to 70 000. At least three age-matched naïve animals were included to verify infectivity of sporozoites during all challenge experiments. Parasitaemia was monitored by daily Giemsa-stained thin blood smears, starting from day 3 after immunization or ANKA-GFP challenge until at least day 17.
