# Supplementary material for: Inactivation of a Plasmodium apicoplast protein attenuates formation of liver merozoites
Source: Mol Microbiol. 2011 Aug 17;81(6):1511–25. doi: 10.1111/j.1365-2958.2011.07787.x (PMC3206223; doi:10.1111/j.1365-2958.2011.07787.x)
Supplement: Supplementary file 1 [file mmi0081-1511-SD1.pdf]

Supporting information for:

Inactivation of a *Plasmodium* Apicoplast Protein Attenuates Formation of Liver Merozoites

Joana M. Haussig, Kai Matuschewski, Taco W.A. Kooij

Contents:

- **Supporting Figure 1**
- **Supporting Tables 1 and 2**
- **Supporting Experimental Procedures**
- **Supporting Reference**

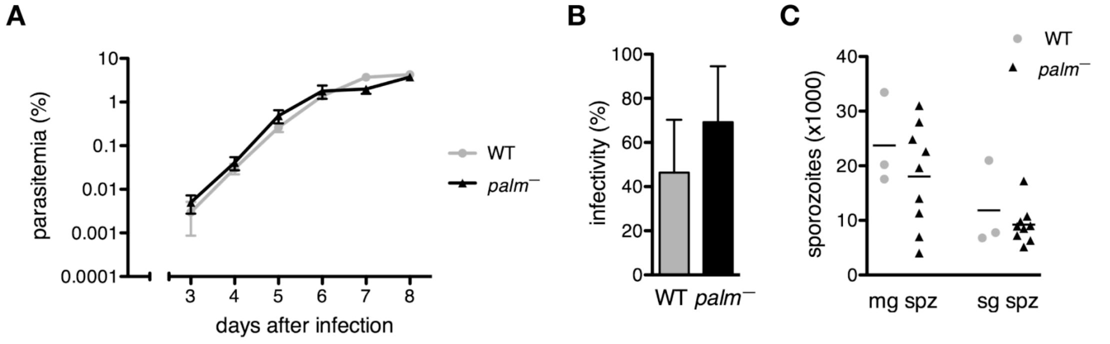

Fig. S1. *palm⁻* parasites display normal life cycle progression during blood stage development and sporogony in the insect vector.

A. Normal blood stage development of *palm⁻* parasites. C57BL/6 mice were injected intravenously with 1,000 blood stage ANKA-GFP or *palm⁻* ANKA-GFP parasites (n=10 each) and monitored for blood stage development of parasites by examination of Giemsa-stained blood-smears. ANKA-GFP and *palm⁻* ANKA-GFP blood stage development is indistinguishable. Shown are mean numbers (± SD).

B. Normal infectivity of *palm⁻* parasites. The number of mosquitoes with oocyst-infected midguts (infectivity) was determined at day 10 after the blood meal. Shown are mean numbers (± SD), for ANKA-GFP (feedings n=3) and for *palm⁻* ANKA-GFP (feedings n=9).

C. *palm⁻* parasites form normal sporozoites in the insect vector. Midgut-associated and salivary gland sporozoites were determined at day 14 and 17, respectively, after the blood meal. Shown are mean numbers per infected mosquito. Number of feedings: ANKA-GFP, n=3; *palm⁻* ANKA-GFP, n=9.

Table S1. Phenotypical analysis of *PALM-mCherry-myc* and three independent *palm*⁻ clones.

		ANKA-GFP	*PALM-mCherry-myc*	*palm*⁻ ANKA-GFP	*palm*⁻ ANKA	*palm*⁻*-NT-tag*
Infectivity[a]	Day 10	46% ± 24	38% ± 6	51% ± 6	33%	57% ± 33
# mg sporozoites[b]	Day 14	23,700 ± 8,500	15,600 ± 7,400	4,700 ± 4,400	28,800	3,300 ± 1,400
# sg sporozoites[c]	Day 17	11,900 ± 7,900	18,600 ± 200	6,100 ± 2,800	58,300	20,400 ± 6,400
		(n=3)	(n=2)	(n=7)	(n=1)	(n=2)
# Liver stages[d]	24h	177 ± 65	139	139 ± 7	188	115
	48h	158 ± 72	152	120 ± 16	152	126
	72h	50 ± 38	41	98 ± 4	111	91
		(n=3)	(n=1)	(n=2)	(n=1)	(n=1)
# Merosomes[e]	Method 1	810 ± 484	NA	22 ± 12	NA	NA
	Method 2	141 ± 16	84 ± 29	NA	1 ± 2	1 ± 2
# Malaria-free mice[f]	Day 6	0/9	0/3 (by bite)	82/82	10/10	20/20
	Day 10	0/9	0/3 (by bite)	62/82	3/10	6/20
	Day 14	0/9	0/3 (by bite)	60/82	3/10	5/20
Prepatency (days)[g]		3	4 (by bite)	8.7	8.4	7.9
# Mice with ECM[h]		9/9	NA	5/20	1/7	0/15
		(n=9)	(n=3)	(n=20)	(n=7)	(n=15)

[a] Numbers of infected midguts were determined at day 10 after the blood meal. Shown are mean numbers (± S.D.).

[b] Midgut-associated (mg) sporozoites were determined at day 14 after the blood meal. Shown are mean numbers (± S.D.).

[c] Salivary gland (sg) sporozoites were determined at day 17 after the blood meal. Shown are mean numbers (± S.D.). N is the number of independent feeding experiments from which the number of mosquito stage parasites was determined.

[d] Liver stages were immunostained with anti-*Pb*HSP70 antibodies at the indicated time points after infection of hepatoma cells. Shown are mean numbers (± S.D.). N is the number of independent experiments.

[e] Numbers of merosomes were counted in a Neubauer chamber 72 hours after infection of hepatoma cells. Shown are mean numbers of triplicate wells (± S.D.). See the methods section for the differences between the two methods used.

[f] Mice immunized twice with 10,000 *palm*$^{-}$ sporozoites were challenged with 10,000 intravenously injected ANKA-GFP sporozoites or in the case of *PALM-mCherry-myc* by natural bite of five infectious mosquitoes. Blood stage infection following challenge was determined by Giemsa-stained thin blood smears at indicated time points.

[g] Prepatency was determined by daily Giemsa-stained thin blood smears. Shown are mean numbers.

[h] Mice were monitored for the development of signature symptoms of experimental cerebral malaria (ECM), such as ataxia, paralysis, convulsions, and coma. N is the number of mice for which prepatency and disease development were monitored.

Table S2. Primer sequences.

Primer Name	Primer Sequence (restriction sites are underlined)
PbPALM-F1	CCAGCCTCAATAGATAAACAGTTC
PbPALM-F2-BamHI	TTTGGATCCTTTTGACGTATGCATTAATCTAGC
PbPALM-F3-NotI	TTTGCGGCCGCTTTTGACGTATGCATTAATCTAGC
PbPALM-F4	TCCTTTGTAATAAAACTTTCTTTGTGG
PbPALM-F5-EcoRI	ATTGAATTCTTAACTCCAACGATGATATAGAGG
PbPALM-F6	AAATGATCCAATATTCTCTTGCAG
PbPALM-F7-HindIII	TTTAAGCTTTTCGCAAAAATATGGATGCAAC
PbPALM-R1-EcoRI	TTTGAATTCTGCCTATATTCTGCCCATAGC
PbPALM-R2	GATGGTTGTGCTGCATTCTG
PbPALM-R3-PshAI	TTAGACATATGTCCATTTGCATTCCTATTATTATCTTCTATG
PbPALM-R4	TTATCAATATCAAAAATGGTCAAATGG
PbPALM-R5-Blunt	AATGGCGGCTAAAATATTTCCATCATGCTTCTTATATATATTATC
PbPALM-R6-KpnI	ATTGGTACCTTACTTTTCCTACCTAAACCTTTTTTG
PbPALM-R7	CCCCCTACATACAGGAGCAG
PbHSP70-F	GCTAACGCAAAAGCAAAGC
PbHSP70-R	TCGGTAAAAGCTACATAGGATG
TgRevPro	CGCATTATATGAGTTCATTTACACAATCC
TgForw	CCCGCACGGACGAATCCAGATGG
mCherryRev	CCCTCCATGTGAACCTTGAAG

Supporting Experimental Procedures

Generation of PALM-mCherry-myc *parasites*

To tag the endogenous PALM for *in vivo* imaging and indirect immunofluorescence assays, we used a replacement strategy. Fragments of the C-terminal coding region and 3' untranslated region (UTR) of *PALM* were amplified from gDNA using gene-specific primers: PbPALM-F5-EcoRI and PbPALM-R5-Blunt (C-terminal fragment, 422 bp), and PbPALM-F7-HindIII and PbPALM-R6-KpnI (3' fragment, 545 bp). Fragments were cloned into a targeting vector, which contains the *mCherry* coding region fused to a quadruple *c-myc* tag sequence and the pyrimethamine-resistant *Toxoplasma gondii* dihydrofolate reductase/thymidylate synthetase cassette.

The resulting plasmid, pPbPALM-mCherry-myc, was linearized with EcoRI and Acc65I and to minimize transfection with partially digested plasmid, the ampicillin resistance cassette was cut with ScaI. Transfection of ANKA-GFP parasites, positive selection, and cloning by limited dilution *in vivo* resulted in a parasite line expressing the endogenous *PALM* fused in-frame via a short spacer sequence to the *mCherry-myc* tag. Correct integration was confirmed using the following specific primer combinations: PbPALM-F4 and mCherryRev (5' integration, 979 bp), TgRevPro and PbPALM-R7 (3' integration, 988 bp), PbPALM-F4 and PbPALM-R6-KpnI (5' ANKA-GFP control, 1450 bp), and PbPALM-F6 and PbPALM-R7 (3' ANKA-GFP control, 906 bp). All primer sequences are listed in Table S2.

Generation of palm^{-} *parasites*

In order to generate knockout parasites, the *P. berghei PALM* locus was disrupted by the standard gene replacement strategy (Janse *et al.*, 2006). Fragments of the 5'UTR and part of the N-terminal coding region and of the 3'UTR were amplified from gDNA using the following primer combinations: PbPALM-F2-BamHI and PbPALM-R1-EcoRI (5' fragment, 587 bp), and PbPALM-F7-HindIII and PbPALM-R6-KpnI (3' fragment, 545 bp). PCR fragments were cloned into the standard transfection vector, which contains the pyrimethamine-resistant *Toxoplasma gondii* dihydrofolate reductase/thymidylate synthetase cassette. The resulting plasmid, pPbPALM-KO, was linearized with BamHI and Acc65I. In addition, the plasmid backbone was cut with SapI to minimize transfection with partially digested plasmid. We transfected *P. berghei* ANKA parasites and a clone, termed ANKA-GFP, which expresses GFP under the control of the constitutive *PbEF1a* promoter (Janse *et al.*, 2006). Recombinant parasites were selected and cloned as described previously (Janse *et al.*, 2006).

For a third independent clone, that we labeled *palm^{-}-NT-tag*, fragments of the 5'UTR including part of the N-terminal coding region corresponding to the apicoplast targeting signal and of the 3'UTR were amplified from gDNA using the following primer combinations: PbPALM-F3-NotI and PbPALM-R3-PshAI (5' fragment, 700 bp), and PbPALM-F7-HindIII and PbPALM-R6-KpnI (3' fragment, 545 bp). PCR fragments were cloned into a targeting vector, which contains the *mCherry* coding region fused to a quadruple *c-myc* tag sequence and the pyrimethamine-resistant *Toxoplasma gondii* dihydrofolate reductase/thymidylate synthetase cassette. The resulting

plasmid, pPbPALM-KO2, was linearized with NotI and ScaI. In addition, the plasmid backbone was cut with SapI to minimize transfection with partially digested plasmid. We transfected, selected and cloned *P. berghei* ANKA-GFP parasites as described previously (Janse *et al.*, 2006).

We generated three independent clones: *palm*⁻ ANKA-GFP and *palm*⁻*-NT-tag* following transfection of the GFP expressing ANKA-GFP line using vectors PbPALM-KO and PbPALM-KO2, respectively, and *palm*⁻ ANKA following transfection of WT ANKA parasites with PbPALM-KO. All clones were phenotypically identical. In our study, the *palm*⁻ ANKA-GFP clone was analyzed in detail. We used the ANKA-GFP strain as a control.

Integration-specific PCR amplification of the *palm*⁻ locus to confirm the predicted deletion of *PALM* in *palm*⁻ ANKA and *palm*⁻ ANKA-GFP parasites was done using the following primers: PbPALM-F1 and TgForw (5' integration, 1261 bp), TgRevPro and PbPALM-R7 (3' integration, 988 bp), PbPALM-F1 and PbPALM-R2 (5' ANKA-GFP control, 799 bp), and PbPALM-F6 and PbPALM-R7 (3' ANKA-GFP control, 906 bp). For 5' ANKA-GFP control of *palm*⁻*-NT-tag* parasites the primers PbPALM-F1 and PbPALM-R4 (904 bp) were used.

Genotyping of ANKA-GFP and *palm*⁻ ANKA-GFP gDNA by Southern blot analysis was performed using the PCR DIG Probe Synthesis kit and the DIG Luminescent Detection kit (Roche), according to the manufacturer's protocol. For amplification of the 5' and 3' probes, primers PbPALM-F2-BamHI and PbPALM-R1-EcoRI, and PbPALM-F7-HindIII and PbPALM-R6-KpnI were used, respectively. The 5' probe was annealed to EcoRI digested gDNA resulting in bands of 8.2 kb (ANKA-

GFP) and 3.6 kb (*palm*⁻ ANKA-GFP). The 3' probe was annealed to KpnI digested gDNA resulting in bands of 1.5 kb (ANKA-GFP) and 5.3 kb (*palm*⁻ ANKA-GFP).

We further confirmed inactivation of *PALM* through RT-PCR. Total RNA was isolated from livers excised from infected mice at 44 hours after infection using the RNeasy Mini kit (Qiagen) following the manufacturer's protocol. To remove contaminating genomic DNA, RNA samples were treated with Turbo-DNA-free (Ambion). After clearance of gDNA contamination was confirmed by PCR, cDNA was synthesized by a two-step PCR reaction using oligo dT primers (Ambion). For the detection of *PALM* transcripts, cDNA samples were tested using gene specific primers PbPALM-F6 and PbPALM-R5 (260 bp). Primers specific for heat shock protein 70 (HSP70, PbHSP70-F and PbHSP70-R, 164 bp) were used to control cDNA load. All primer sequences are listed in Table S2.

Supporting Reference

Janse, C.J., Franke-Fayard, B.M.D., Mair, G.R., Ramesar, J., Thiel, C., Engelmann, S., *et al.* (2006) High efficiency transfection of *Plasmodium berghei* facilitates novel selection procedures. *Mol Biochem Parasitol* **145:** 60-70
